# Leishmaniasis in the Middle East: Incidence and Epidemiology

**DOI:** 10.1371/journal.pntd.0003208

**Published:** 2014-10-02

**Authors:** Nasir Salam, Waleed Mohammed Al-Shaqha, Arezki Azzi

**Affiliations:** 1 Department of Biochemistry, College of Medicine, Al-Imam Mohammad Ibn Saud Islamic University (IMSIU), Riyadh, Saudi Arabia; 2 Department of Pharmacology, College of Medicine, Al-Imam Mohammad Ibn Saud Islamic University (IMSIU), Riyadh, Saudi Arabia; New York University, United States of America

## Abstract

Leishmaniasis is a major health problem worldwide, with several countries reporting cases of leishmaniasis resulting in loss of human life or a lifelong stigma because of bodily scars. The Middle East is endemic for cutaneous leishmaniasis, with countries like Syria reporting very high incidence of the disease. Despite several countries establishing national control programs for containing the sandfly vector and treatment of infection, the disease continues to spread. In addition to the endemicity of the region for leishmaniasis, the Middle East has seen a great deal of human migration either for earning of livelihood or due to political upheaval in the region. These factors contribute to the spread and proliferation of the causative species *Leishmania* and its sandfly host. This review discusses the current epidemiological scenario in Iraq, Syria, Saudi Arabia, and Jordan, emphasizing the number of cases reported, vector species, *Leishmania* species, and treatment available. The data is primarily from WHO reports for each country and current and old literature.

## Introduction

Parasitic diseases of the genus *Leishmania* are a huge burden on human health and society. Their incidence is found to be prevalent in some of the poorest countries in the world, and they gain lesser attention than other infectious diseases like malaria, tuberculosis, and AIDS. They are categorized as a neglected tropical disease because although they cause significant mortality, there is little effort on the part of the global community and pharmaceutical industry to invest in research and development of better and innovative therapeutics because of a lack of sufficient incentives. Also, the disease is found primarily among the poorest of the poor, who have no influence over policy-makers and little access to healthcare [1]. According to recent reports, Leishmaniasis is endemic in 98 countries, and around 1.3 million new cases are reported every year, with an estimated 20,000 to 40,000 deaths every year [2].

It is spread through the bite of female phelobotomine sandflies by nearly 20 different species of *Leishmania*. Leishmaniasis can manifest itself in different forms; depending upon the infecting species of *Leishmania*, the disease could emerge as cutaneous, mucocutaneous, or visceral leishmaniasis [3]. Cutaneous leishmaniasis (CL) is the most common and least fatal form of the disease, identified by ulcerative skin lesions, and it is caused by *Leishmania major, L. tropica, L. aethiopica, L. mexicana, L. braziliensis, L. guyanensis, L. panamensis, L. peruviana*, and *L. amazonensis*. Almost two-thirds cases of CL are reported from six countries: Afghanistan, Algeria, Brazil, Colombia, Iran (Islamic Republic of), and the Syrian Arab Republic [4]. Mucocutaneous leishmaniasis results in the complete or partial destruction of mucous membranes of the nose and mouth, and it is caused by *L. braziliensis*, and *L. panamensis*. The most life-threatening form of the disease is visceral leishmaniasis (VL), in which the pathogen disseminates to internal organs like the liver, spleen, and bone marrow. It is caused by *L. donovani* and *L. infantum* (known as *Leishmania chagasi* in South America). Clinical symptoms of visceral leishmaniasis generally include prolonged and irregular fever associated with chills, hepatosplenomegaly, lymphadenopathy, progressive anemia, weight loss, and hypergammaglobulinemia. More than 90% of visceral leishmaniasis cases are reported from Bangladesh, Brazil, Ethiopia, India, Sudan, and South Sudan [3].

## Methodology

The data presented in this review are gathered primarily from WHO reports for each country and from an extensive literature search on PubMed using the term “Leishmaniasis” followed by the name of each endemic country. Similar searches were carried out using the term “sandfly”, “Leishmaniasis epidemiology,” and “Leishmaniasis Middle East”.

## Host–Pathogen Interaction in Leishmaniasis

Despite differences in the causative species and clinical symptoms, there are commonalties in all infections, and these commonalities are persistent parasitism, macrophage as the host cell, and host immunity determining the outcome of infection [5]. The digestive tracts of female sandflies are the home for extracellular promastigotes, which survive by attaching to epithelium of the insect gut. These promastigotes are injected under the skin of the human host during a blood meal. Tissue resident macrophages, neutrophils, Langerhans cells, and keratinocytes are the cells with which promastigotes interact [6]. The parasites are rapidly phagocytosed by the macrophages directly or through apoptosing neutrophils through a complement receptor (CR3) dependent mechanism. The complement protein C3b binds the *Leishmania* surface glycoprotein gp63 and promotes phagocytosis of the parasite. Inside macrophages, *Leishmania* resides in the parasitophorous vacuole and transforms into amastigote form. Like other intracellular pathogens, *Leishmania* avoid degradation by inhibiting the fusion of the phagosome with hydrolase-rich endocytic organelles through lipophosphoglycan (LPG) mediated disruption of lipid microdomains in the phagosomal membrane, thus impairing macrophage-based defensive mechanisms [7]. Additionally, inside the phagosome *Leishmania* produces a surface acid phosphatase that inhibits oxidative burst and is an active proton pump that keeps the phagosomal pH close to neutral [8]. Infection leads to the up-regulation of suppressive signaling pathways, and modulates intracellular phosphatases and kinases. *Leishmania* avoid detection by CD8 T cells by down-regulating MHC II expression on the surface of infected macrophages. Together, these cellular events lead to the unresponsiveness of infected macrophage to cytokine, and inhibition of leishmanicidal mechanisms like production of reactive nitrogen and oxygen intermediates [8]. Apart from affecting macrophages *Leishmania* also down-regulates dendritic cell-based immune responses by inhibiting migration, maturation, antigen presentation abilities, and the production of IL-12. Effects on dendritic cells have a direct bearing on the development of successful Th1 response, required to clear the infection. There is no clear evidence for the existence of either a net Th1 or Th2 response in the human host. While infections with *L. major* result in self-healing lesions and lifelong immunity, infections with *L. donovani* lead to subclinical infection, resulting in protective immunity or, in clinical cases, fatal outcomes if not treated. Development of protective immunity appears to be dependent on the infecting species, host genetic factors, and the size of the inoculum [3], [7], [9].

## Diagnosis and Therapy in Leishmaniasis

Diagnosis of leishmaniasis is based on endemicity, clinical symptoms, and laboratory test results. Often diseases of different etiology but similar symptoms like schistosomiasis and malaria are present in the same endemic region as leishmaniasis; therefore, careful differential diagnosis is very critical for epidemiological surveys and therapeutic purposes [10]. Microscopic examination of Giemsa-stained parasites from skin lesion and aspirates from spleen, bone marrow, and lymph node for CL and VL, respectively, are commonly used. Serological test based on the detection of anti-*Leishmania* antibodies in patient serum and agglutination of the parasite is also used. Another method is the detection of antibodies against a 39 amino acid repeat sequence of a Kinesin-like protein, present in the amastigotes and highly conserved among species causing VL, which in a dipstick format shows 95% to 100% accuracy in VL patients irrespective of geographical location [11], [12]. However, PCR-based methods are considered to be more specific and can detect current infection, unlike immunological tests that cannot distinguish between current and past infections. A major deterrent for use of PCR-based methods is that it requires a moderate setup of instruments and expertise that is not available outside research settings in many resource-poor countries. Additionally, health agencies must not only allocate funding for the cost of diagnostics itself but also bear the cost of procurement of reagents and hiring and training of sufficient laboratory staff [13]. Apart from the problems with availability and cost-effectiveness of the technique, there are no clear criteria to distinguish cases from non-cases, and lack of standardized protocols makes it challenging to use this technique extensively in the field [10]. Pentavalent antimonials are the drug of choice and have been in use for more than 70 years now. Sodium stibogluconate and meglumine antimoniate are widely used for treatment of all kinds of leishmaniasis. Both these drugs are very toxic and can have serious side effects that include cardiac arrhythmia and pancreatitis, and their use could lead to life-threatening situations. Additionally, there is widespread emergence of drug resistance due to non-standard intake and misuse of the drug. Amphotericin B, Miltefosine, Paromomycin, and sitamaquine are other drugs that are gradually replacing antimonials, either given alone or in combination with them [12], [14]. CL, which is the predominant form of the disease found in the Middle East, can also be treated using topical medication. Paromomycin, an aminoglycoside antibacterial, is also effective against leishmaniasis. Different formulations have been tried with varied degrees of success [15]. Recently, a formulation of 15% Paromomycin or a combination of 15% Paromomycin and 0.5% gentamycin was able to achieve a cure rate of 81% in a study comprising 375 patients, indicating no added benefit of gentamycin and no severe side effects [16].

## Vector Distribution and Behavior

Different species of sandfly show different distribution and seasonal variation, but in general, they survive at temperatures above 16°C, up to 44°C, and are mostly found between the months of May to November, showing maximum activity on warm, clear nights with low wind speed. Certain species, like *Phlebotomus bergeroti*, *P. papatasi*, and *P. arabicus*, prefer indoor habitats, while others, like *P. alexandri*, are found in outside environment [17], [18]. Sandflies are generally no more than 3.5 mm in length and covered with dense hair, holding their wings in a characteristic V-shaped position. Both male and female adults survive on sugary secretions from plants; females, though, require blood meal for development of egg batches. They are generally active during the night and early morning and characteristically hop across the skin to find a blood meal. The eggs are deposited in batches in warm and moist places close to the larval food sources [18], [19]. There are four different modes of transmission of leishmaniasis in the Middle East zoonotic cutaneous leishmaniasis (ZCL), caused by *L. major*, transmitted through *P. papatasi*, with rodent species of *Psammomys obesus, Meriones libycus*, *Nesokia indica*, and *Rhombomys opimus* serving as nonhuman reservoirs. Zoonotic visceral leishmaniasis (ZVL) is caused by *L. infantum*, spread through *P. galilaeus, P. syriacus, P. tobbi, P. halepensis*, and the dog species of *Canis familiaris* acts as nonhuman reservoirs. Anthroponotic cutaneous leishmaniasis (ACL), caused by *L. tropica* and spread through *P. sergenti*, circulates exclusively in humans. Anthroponotic visceral leishmaniasis (AVL) caused by *L. donovani* spreads through *P. alexandri* without any non-human reservoir [19], [20], [21].

Due to the absence of a vaccine and emergence of drug resistance, leishmaniasis continues to be a burden on society. Additionally, with human migration increasing, there is a fair chance that infectious disease could spread to other areas, introducing the pathogen to newer environments and leading to mutations and emergence of more virulent strains. Under these circumstances, there is an increasing pressure for the development of novel vaccines, therapeutic targets, and biomarkers of infection [16], [22]. There is also an urgent need to report and document cases of leishmaniasis from endemic and non-endemic regions that can give government and health agencies an idea of the prevalence, disease-causing species, vector, nonhuman reservoir, and efforts to control the infection [23]. This review summarizes incidence and epidemiology of leishmaniasis in Iraq, Syria, Saudi Arabia, and Jordan.

## Leishmaniasis in Iraq

**Figure 1:**
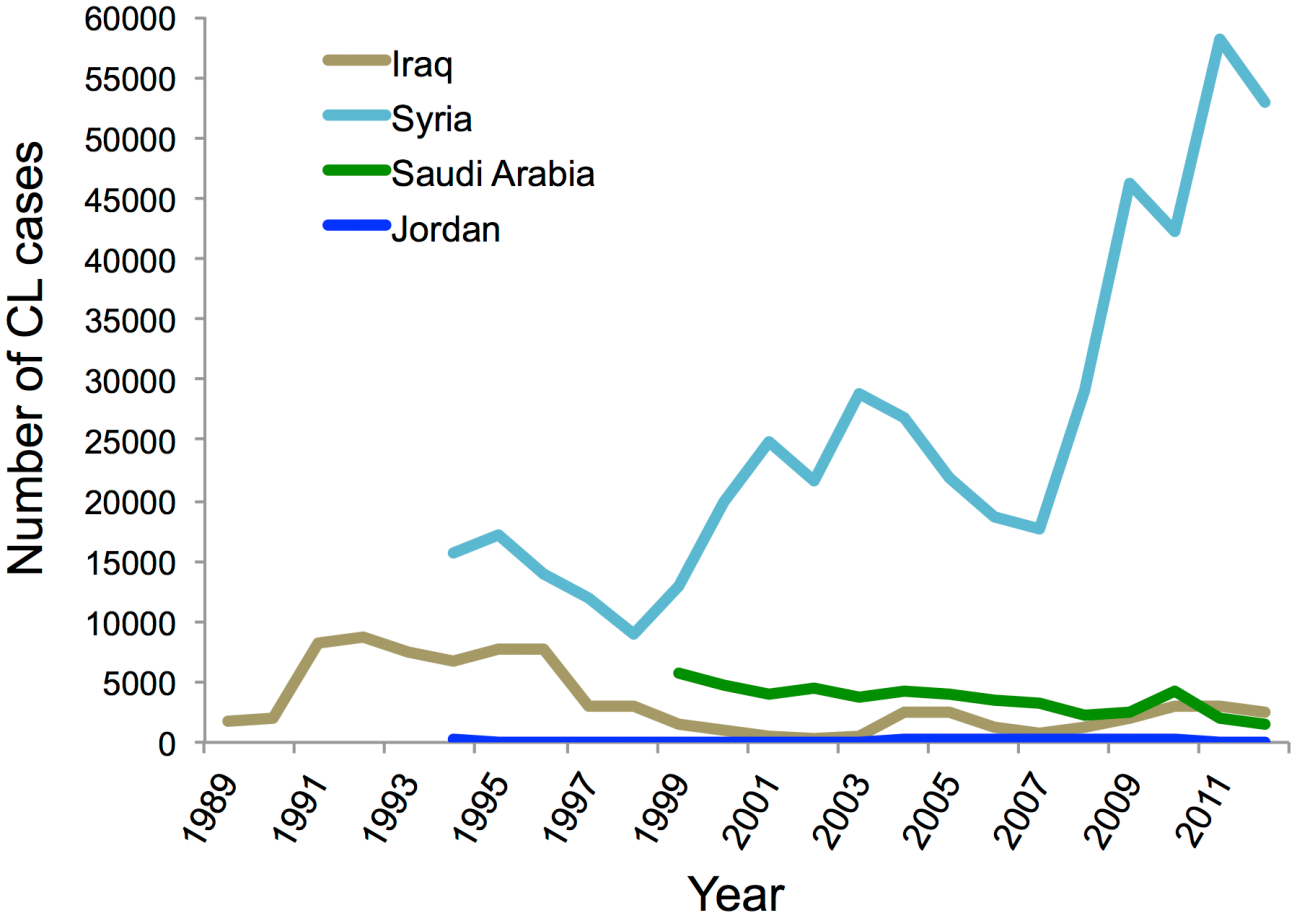
Year-wise trend of CL cases reported in Middle East. Data is based on WHO reports for each country [48].

**Figure 2:**
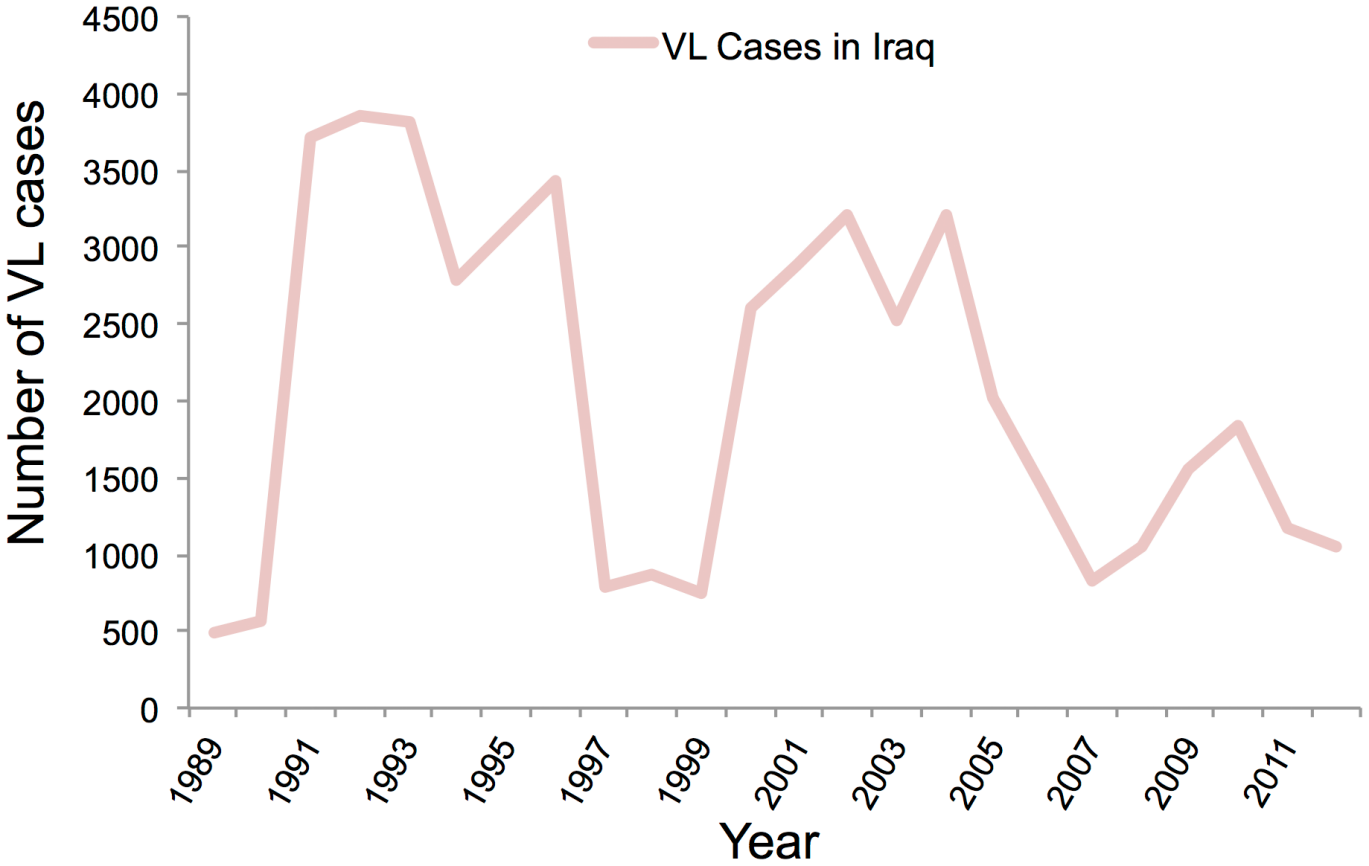
Year-wise trend of VL cases reported in Iraq. Data is based on WHO estimates.

Leishmaniasis is endemic in Iraq, where both forms of the disease, cutaneous and visceral, are found. Iraq, with a population of nearly 32 million, where 23% are living below the national poverty line, has seen much strife and struggle in the past 25 years. Maximum number of cases of Leishmaniasis were reported in these early years of war and population displacement; in 1992 the number peaked at 45.5 cases per 100,000 of population [24]. Poor sanitation, movement of non-immune population to endemic areas, and a rise in vector population were the reasons for this increase in leishmaniasis cases. However, by 2004 the cases of leishmaniasis were declining, but have seen a sudden rise in the past couple of years (Figure 1). Generally, the data up to 2012 are available. Because of the lack of proper diagnostic facilities and low priority of leishmaniasis, it has been severely underreported. Clinicians generally rely upon clinical symptoms along with serological test for the diagnosis [25]. Visceral leishmaniasis is generally found in central Iraq but has extended to southeastern parts also, after the Gulf War. *L. donovani* and *L. Infantum* are the causative agents, with 90% of the cases reported in children under five years of age, and it is spread through the *P. alexandri* species of sandfly [26]. There were 1,049 cases of VL reported in 2008 and 1,045 in 2012, which came down from 3,218 cases in 2004 because of the efforts of the Iraqi health ministry and WHO (Figure 2). Most of these cases were reported from the eastern provinces of Diala, Wasit, Missan, and Basrah [27]. CL, however, is quite prevalent in the entire country except for three northeastern provinces, and it is spread by *P. sergenti* and *P. papatasi* species of sandfly with *L. major* and *L. tropica* as the causative agents. In 2008 there was an outbreak of CL, with 300 cases being reported from Diwania and 400 cases reported in 2009 from Rahmania province (Figure 1). Pentavalent antimonials are the generally prescribed treatment, with 90% cure rate seen for VL patients and 80% for CL patients [28].

## Leishmaniasis in Syria

**Figure 3:**
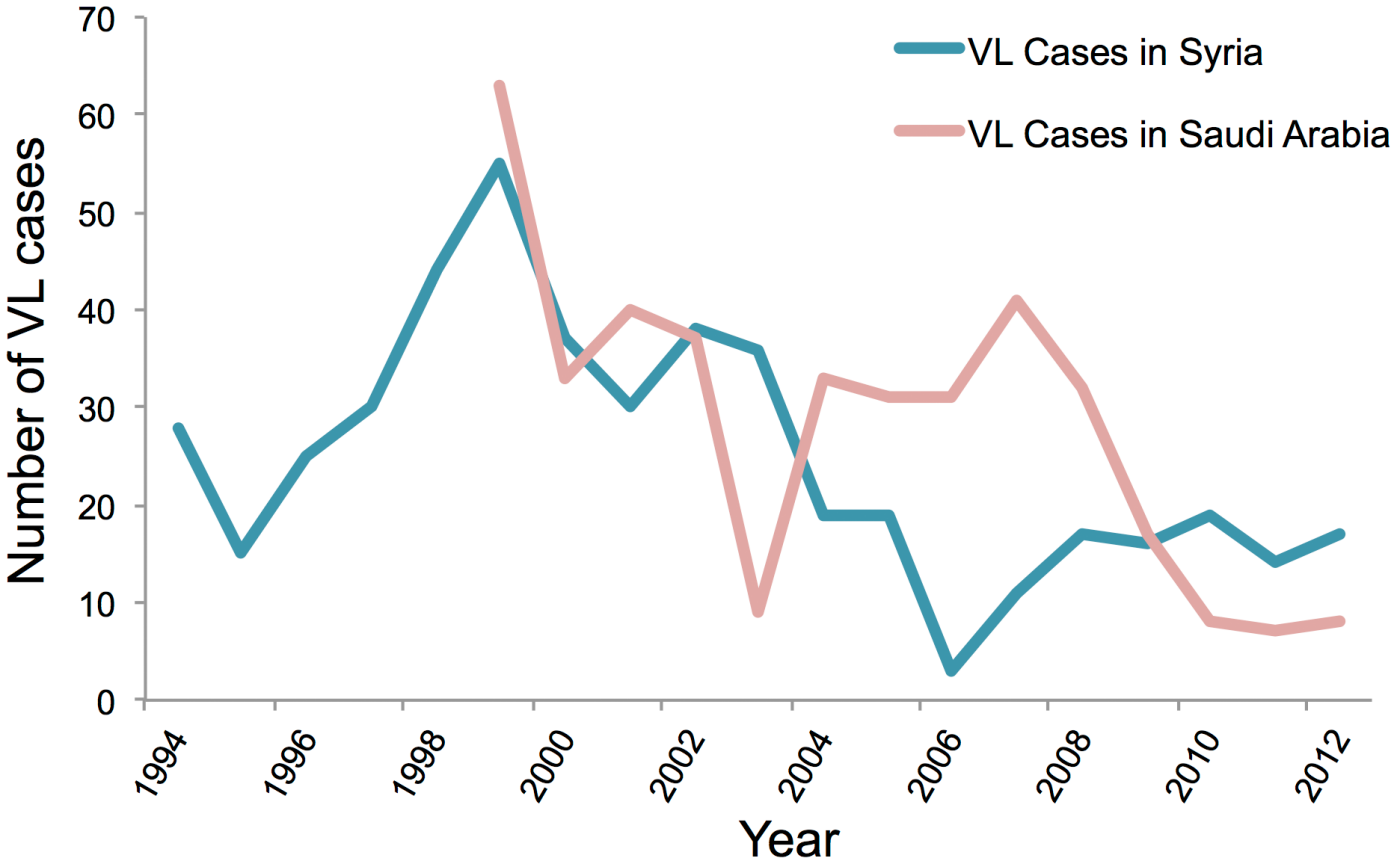
Year-wise trends of VL cases reported in Syria and Saudi Arabia. Data is based on WHO estimates.

Cases of CL have been endemic in the Aleppo region in Syria for a very long time, with the first occurrence being reported as far back as 1745 when it was commonly referred to as “Aleppo boil” [29]. Antimalarial spraying in the 1950s led to fewer reported cases of leishmaniasis; an estimated 217 cases per year were reported from 1962 to 1971. However, there has been a gradual increase in the number of cases reported from the 1990s onwards, with a maximum number of cases of 58,156 being reported in 2011 from Idlib, Hamah, and Halab provinces (Figure 1). *L. tropica* and *L. major* are the primary causative *Leishmania* species with *P. sergenti* and *P. papatasi* species of sandfly serving as the vector [30]. Syria, with nearly 20 million people, where 37% are below 14 years of age, has seen great political turmoil in the past couple of years, and this has led to huge population displacement. Concerns about sanitation and waste disposal are growing. Public health infrastructure is in shambles, and there is a severe lack of health workers. These additional factors have led to a surge in new CL cases being reported (Figure 1) with nearly 52,983 cases being reported in 2012. The cases of visceral leishmaniasis are few and far between, with only 19 cases being reported in 2010 (Figure 3). The primary agent of VL spread is *L. infantum*. Diagnosis is based on clinical manifestation of the disease and skin lesions in case of CL and serodiagnostic tests and culturing of parasites for VL. Pentavalent antimonials continue to be the preferred mode of treatment with 85%–90% cure rate being achieved for CL and 100% cure rate for VL [31].

## Leishmaniasis in Saudi Arabia

**Figure 4:**
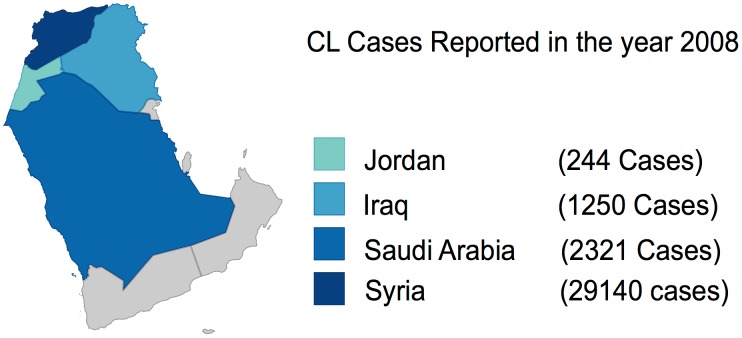
Relative distribution of CL cases reported in the year 2008.

**Figure 5:**
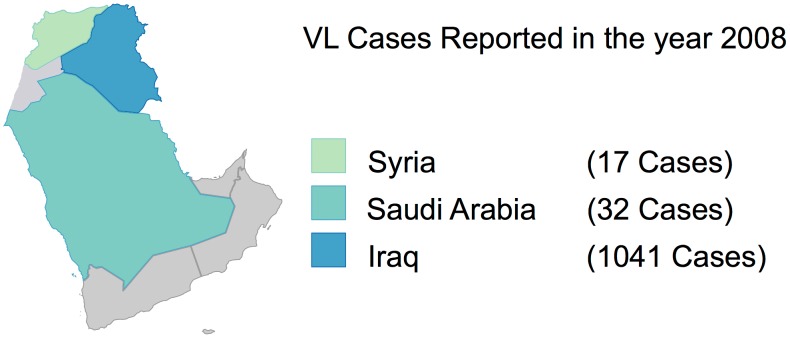
Relative distribution of VL cases reported in the year 2008.

CL is the most common form of the disease present in Saudi Arabia and is commonly known as okhet, dommal, nafra, and El-mohtafara. The disease is prevalent in Al-Hassa oasis, and reached epidemic proportions in 1983 with 18,000 cases of CL being reported but subsided later on after a National Control Program was set up [32], [33]. Initial rise in disease was due to rapid urbanization and large-scale immigration from other countries to Saudi Arabia. The species causing the disease is *L. major* in central and eastern provinces and *L. tropica* in west and southwest provinces. The main vector of the disease is *P. sergenti*, and the disease affects males and females equally [34], [35]. The disease mostly affects patients of 15–44 years of age and generally effects extremities; most patients have a single lesion, with less than 5% showing multiple lesions on hands, legs, and face. According to WHO, 4,753 cases were reported in 2006, but since then, prevalence has been gradually declining, with 2,549 cases being reported in 2009 (Figure 1). Incidences of VL are sporadic in nature with only eight cases reported in 2012 (Figure 3) and found primarily in Jazan region. *L. tropica* and *L. donovani* are the causative species with *P. sergenti* serving as the carrier. Saudi Arabia has been able to control leishmaniasis, with a gradual decline in the number of cases being reported (Figure 4). The country has an active national control program, which carries out case detection in focus areas where one or more cases are found (Figure 5). Additionally, there are vector control programs and reservoir control programs to further control insect and nonhuman reservoirs. Diagnosis is based on clinical manifestation and microscopic examination of skin lesion samples in cases of CL, while VL is confirmed by microscopic examination of bone marrow and spleen aspirates. Pentavalent antimonials are readily administered, with 95% cure rate being achieved for CL and 98% cure rate achieved for VL [36].

## Leishmaniasis in Jordan

Like other countries in the Middle East, CL is endemic in Jordan, with the first case being reported back in 1929. Around 524 cases were reported between 1973 and 1978, with most of them being reported from Jordan valley, which represents the endemic region of CL in Jordan [37], [38], [39]. In Swaimeh region of Jordan valley, which is considered to be hyperendemic, 100% positivity was reported in 1992 for CL based on *Leishmania* skin tests in individuals over five years of age. More males (72.4%) are infected than females (27.6%), and in the majority of cases skin lesions are present on face and neck as compared to hands, arms, and legs. The major causative species is *L. major*, which is responsible for 75% of the cases and is spread through *P. papatasi*; cases from *L. tropica* are few and are reported from the northern region of Jordan. Outbreaks of CL have been reported (Figure 1) in Aqaba, north Agwar, and south Shuneh, with 100–200 cases being reported from 2004 to 2008 [40]. However, according to Mosleh et al., there has been severe underreporting of the cases by an estimated factor of 47 times, which makes it really difficult for health agencies and WHO to work towards its eradication [41]. VL is rare in Jordan, with only 15 cases being reported since 1960, and it is caused by *L. infantum* [42]. Despite Jordan valley being an endemic region for CL, there is no national control program for leishmaniasis. Diagnosis is based on examination of skin lesion for CL and microscopic examination of aspirates in specialized hospitals only. More advanced techniques like PCR are not available within Jordan; there is a shortage of trained medical personnel for treatment of CL and a lack of public awareness for this disease. More traditional methods are used for its treatment in rural areas, and while hospitals do provide *Leishmania*-specific treatment, drug shortages have been reported in the past. Intralesional and systemic doses of pentavalent antimonials have reported 100% cure rate [43]. Jordan, with a population of more than 6 million people, where 13.3% are living under the national poverty line, needs to prioritize the treatment of leishmaniasis.

## Discussion

Among the factors that potentiate the incidence of parasitic diseases, poverty, rapid urbanization, and human migration due to violent conflicts are often cited as the most important determinants of epidemiological outbreaks. Primary effects of poverty are mediated through lack of access to health care and compromised nutritional status putting the exposed population at greater risk of the disease. Rapid urbanization leads to the settlement of rural populations in makeshift surroundings without proper sanitation and housing conditions, leading to a greater exposure to human or animal carriers and a rise in vector population, resulting in disease outbreaks. Civil wars result in migration of a large number of non-immune populations to endemic regions or introduction of exotic diseases to newer surroundings, resulting in epidemics [44]. These factors were at play during the civil war in South Sudan, where 100,000 people died due to visceral leishmaniasis between 1984 to 1994 [45]. Iraq has also witnessed some of the worst violence and strife in the past few years, resulting in the loss of momentum of a credible healthcare system, which is on its way back to recovery [46]. However, we are witnessing some of these drivers of leishmaniasis in the conflict-prone region of Middle East, especially in Syria (Figure 4). According to reports, close to 100,000 people were infected with CL in the past 2 years because of a breakdown in health services and garbage collection, which might have led to an increase in sandfly numbers. Overall, 55% of public hospitals are damaged and 142 health care workers have been directly affected due to death, injuries, or kidnapping [47]. Many Syrian refugees are taking shelter in neighboring countries of Jordan, Lebanon, and Iraq, and are affected disproportionately by leishmaniasis. The increase in disease rates can be clearly linked to breakdown of basic health care infrastructure and population displacement. Efforts to provide relief in war zones are often impeded by local violence endangering the life of care providers. These are trying times for a region where leishmaniasis is already endemic; a concerted effort is required from the global community for the control of the disease. WHO member countries have pledged US$1.5 billion to deal with the humanitarian crisis in Syria; Saudi Arabia has donated nearly US$2 million to provide essential medicine, vaccines, and medical equipment [47]. An active and alert global community could undo damages done to human health due to political instability, violence, and conflicts. Global efforts through governmental and nongovernmental cooperation in the past have resulted in the eradication of fatal and debilitating diseases like smallpox and polio. Similar strategies are required for the control and elimination of leishmaniasis.

## Conclusion

Middle Eastern countries are at greater risk from leishmaniasis because this region is endemic for CL and sees a great deal of human migration from other parts of the world. Countries with ample resources, like Saudi Arabia, have taken good measures to control the disease, evident by a gradual decline of cases reported in the past few years; however, other countries, like Syria and Iraq, should put an emphasis on health care facilities for the control of leishmaniasis. A massive effort on reservoir and vector control along with actively pursuing diagnosis in endemic foci will be helpful. Additionally, proper and studious reporting of cases is a necessity for the eradication of this disease, as health care practitioners rely on these data for framing health policies, and there are cases of severe underreporting from some parts of the Middle East. Additionally, drug resistance to pentavalent antimonials and their serious side effects reckons for a greater push towards vaccine research and better therapeutics.

**Top Five Papers**

1. Jacobson RL (2011) Leishmaniasis in an era of conflict in the Middle East. Vector Borne Zoonotic Dis 11: 247–258.
2. Majeed B, Sobel J, Nawar A, Badri S, Muslim H (2012) The persisting burden of visceral leishmaniasis in Iraq: data of the National Surveillance System, 1990–2009. Epidemiol Infect: 1–4.
3. Tayeh A, Jalouk L, Cairncross S (1997) Twenty years of cutaneous leishmaniasis in Aleppo, Syria. Trans R Soc Trop Med Hyg 91: 657–659
4. Al-Tawfiq JA, AbuKhamsin A (2004) Cutaneous leishmaniasis: a 46-year study of the epidemiology and clinical features in Saudi Arabia [1956–2002]. Int J Infect Dis 8: 244–250.
5. Mosleh IM, Geith E, Natsheh L, Abdul-Dayem M, Abotteen N (2008) Cutaneous leishmaniasis in the Jordanian side of the Jordan Valley: severe under-reporting and consequences on public health management. Trop Med Int Health 13: 855–860.

**Key Learning Points**

- There is a substantial rise in cases of cutaneous leishmaniasis in some parts (Syria) of the Middle East.
- Incidences of visceral leishmaniasis are sporadic in nature and have been reported mostly from Iraq in the past.
- There has been severe underreporting of leishmaniasis cases in some countries, resulting in lack of preparedness to deal with its growing incidences.
- Rapid urbanization and human migration in this region have lead to the spread of leishmaniasis and its sandfly vector.
